# Ruptured Cornual Ectopic Pregnancy: A Rare and Challenging Obstetric Emergency

**DOI:** 10.7759/cureus.47842

**Published:** 2023-10-27

**Authors:** Chirag Sharma, Hina Patel

**Affiliations:** 1 Obstetrics and Gynaecology, GMERS (Gujarat Medical Education & Research Society) Medical College and Hospital, Valsad, IND

**Keywords:** high-risk obstetrics, ruptured cornual ectopic pregnancy, obstetric hysterectomy, emergency obstetric care, cornual ectopic pregnancy

## Abstract

Cornual pregnancy is an infrequent form of ectopic pregnancy characterised by the implantation of the embryo at the intersection between the fallopian tube and the uterus. The incidence of ectopic pregnancy is higher in the ampullary region of the fallopian tube. Nevertheless, cornual (interstitial) pregnancy is observed in approximately 2-4% of cases involving ectopic pregnancies. A cornual gestation is considered to be a highly perilous and potentially life-threatening form of ectopic pregnancy, with a mortality rate that is two to five times more than that of other types of ectopic pregnancies. Due to the myometrium's capacity for stretching, the presentation of these cases typically occurs at a later stage, typically between seven and 12 weeks of gestation. Haemodynamic instability is typically observed in patients with ruptured cornual ectopic pregnancy. This study presents a case of a 40-year-old woman, G5P4L1D3, who arrived at the labour room of GMERS (Gujarat Medical Education & Research Society) Medical College and Hospital, Valsad, experiencing shock at eight weeks of gestation. Based on the clinical examination and ultrasound report, a preliminary diagnosis of ruptured cornual ectopic was established. The patient was resuscitated followed by an emergency laparotomy as a critical intervention to preserve their life. The primary approach for addressing maternal mortality caused by cornual pregnancy involves early detection and intervention.

## Introduction

Cornual pregnancy is an infrequent kind of ectopic pregnancy characterised by the implantation of the embryo at the confluence of the fallopian tube and the uterus [1,2].

The occurrence of ectopic pregnancy is higher in the ampullary region of the fallopian tube. Nevertheless, cornual (interstitial) pregnancy is observed in approximately 2-4% of ectopic pregnancies [3]. This particular type of ectopic pregnancy poses a higher level of risk compared to other forms, as it has the potential to result in severe haemorrhage, shock, and uterine rupture. The associated mortality rate for this condition is from 2% to 2.5% [3].

There is ongoing controversy regarding the precise description of cornual ectopic pregnancy. Most of the authors recognise ‘interstitial’ and ‘cornual’ as synonyms. Nevertheless, certain individuals employ the term 'cornual' to describe pregnancies that occur in a uterus with a bicornuate or septate structure [4,5]. A cornual pregnancy is characterised by the occurrence of implantation and subsequent growth of a gestational sac within the upper and lateral regions of the uterus, as per its established definition. On the other hand, an interstitial pregnancy refers to the implantation of a gestational sac within the proximal, intramural segment of the fallopian tube, which is surrounded by the myometrium [6,7]. The region in question has a significant degree of vascularity, which consequently increases the susceptibility to haemorrhaging [8]. This particular location is situated within the segment of the fallopian tube that traverses the muscular stratum of the uterus. The measured dimensions of the object are roughly 1-2 cm in length and 0.7 cm in width. Its blood supply is derived from Sampson's artery, which is anatomically linked to the ovarian and uterine arteries [9,10]. From a pathogenic perspective, the primary risk factor of utmost significance is the compromised functionality of the fallopian tubes. Various factors that contribute to compromised tubal function, such as persistent pelvic inflammation, endometriosis, or tubal surgery, are associated with an elevated likelihood of experiencing an ectopic pregnancy [11].

Cornual pregnancies tend to experience rupture at a later stage compared to other tubal pregnancies due to the greater distensibility of the myometrium. Due to this factor, the mortality risk associated with this type of ectopic pregnancy is two to five times higher compared to other cases [12,13]. Diagnosing an interstitial ectopic pregnancy prior to rupture poses a challenge; yet, timely identification and management of this illness are imperative to mitigate the associated morbidity and mortality risks.

## Case presentation

A patient, aged 40 years, who has had five pregnancies, four live births, and three previous pregnancies ending in foetal loss, arrived at the labour department during the 8th week of her pregnancy, as determined by the estimated date of conception. She reported experiencing abdominal pain for a duration of six hours. At the time of examination, the individual exhibited consciousness and orientation. The patient exhibited a lack of fever, along with a pulse rate of 136 beats per minute and blood pressure measuring 90/50 mmHg. Additionally, the patient's oxygen saturation level was recorded at 89% (Table 1).

**Table 1:** Vital signs at initial presentation

Vital signs	Result
Pulse	136/min
Blood pressure	90/50 mmHg
Respiratory rate	22/min
Pulse oximetry (SpO_2_)	89%
Temperature	36.9°C

The individual had indications of significant dehydration accompanied by pronounced pallor. During the abdominal examination, the presence of widespread discomfort throughout the abdomen was seen. The pelvic examination yielded findings of a retroverted uterus accompanied by bilateral fornical discomfort, as well as cervical motion tenderness. The patient did not have prior ultrasound reports confirming her pregnancy. The result of her urine pregnancy test done at our institute indicated a positive outcome. The patient was diagnosed with a ruptured ectopic pregnancy based on clinical findings and an ultrasound report, which was suggestive of an approximately 8.4 x 8.3 x 6.7 cm size heterogenous lesion in the right adnexal region with moderate free fluid noted in Morrison's pouch, perihepatic, perisplenic, hepatic and splenic flexure, and pelvic region. Subsequently, the patient underwent laparotomy following initial resuscitation measures. The patient's preoperative laboratory results indicated a haemoglobin level of 4.6 gm%, a B-positive blood group, and an abnormal coagulation profile (Table 2).

**Table 2:** Laboratory test findings

Laboratory test	Result
Haemoglobin	4.6 g/dl
Total WBC count	23,300/mm3
Platelet	312,000/mm3
Prothrombin time	24 seconds
International normalized ratio	1.8
Blood urea nitrogen (BUN)	27 mg/dl
Serum creatinine	0.8 mg/dl
Potassium	3.8 mmol/L
Sodium	139 mmol/L
Urine human chorionic gonadotropin	Positive

**Figure 1:**
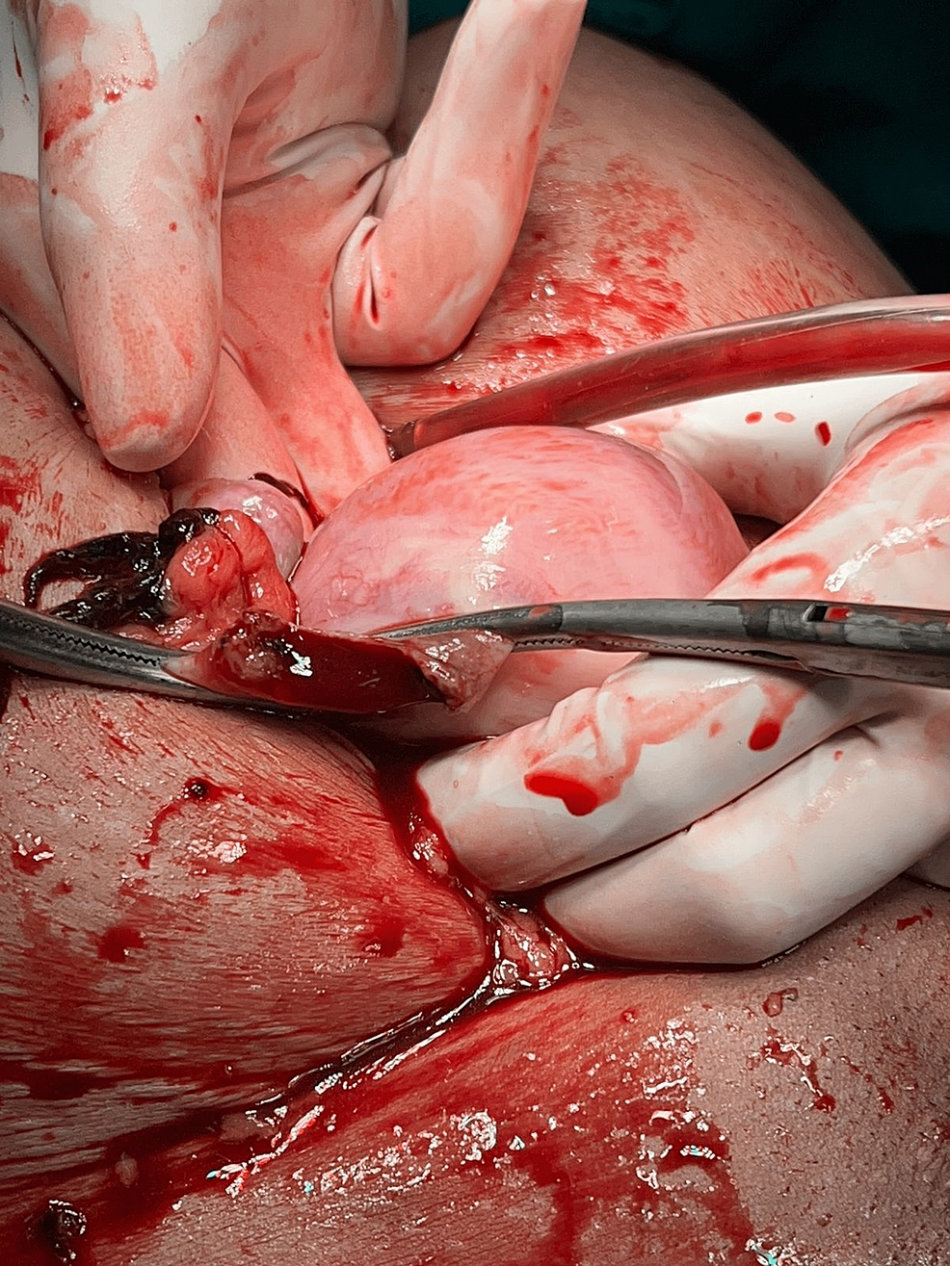
Right ruptured uterine cornu (clamped)

The patient's obstetric history includes a full-term normal vaginal delivery that occurred 18 years ago, resulting in the birth of a healthy male child who is still living. Additionally, the patient experienced three preterm vaginal deliveries in the preceding nine, six, and three years, respectively, all of which unfortunately resulted in neonatal death. Prior to performing the laparotomy, the attendant obtained informed consent. Additionally, arrangements were made for the administration of four units of packed red blood cells (PRBC), four units of fresh frozen plasma (FFP), and two units of platelets. The surgical procedure of emergency laparotomy was conducted under the administration of general anaesthesia while adhering to universal precautions. Approximately 1100 millilitres of blood, together with 440 g of blood clot, were extracted upon the incision of the abdominal cavity. The uterus, fallopian tubes, and ovaries were exteriorized, revealing a right-sided ruptured uterine cornu accompanied by torrential bleeding (Figure 1).

The decision to proceed with a hysterectomy was made, and informed consent was obtained. An emergent total abdominal hysterectomy procedure was performed, as there was profuse bleeding from the right-sided ruptured uterine cornu. Full haemostasis was successfully established, a drain was inserted, and the abdomen was closed in layers following a mop and instrument count. Following the surgical procedure, the patient was monitored in the ICU for a duration of seven days. Throughout the course of her treatment, a cumulative amount of four units of PRBC, four units of FFP, and two units of platelets were administered via transfusion. The patient received ventilatory support in addition to inotropic assistance until the third day after the operation. The patient underwent the process of weaning on the fourth day after the surgery and was subsequently transitioned to non-rebreather mask (NRBM) oxygenation mode with a flow rate of 10 litres per minute. The dressing and removal of the urinary catheter and drain were performed on the fifth day following the surgical procedure. The patient was transferred to the high-dependency unit (HDU) on the eighth day following the surgical procedure. The patient was discharged from the hospital on the 12th day following the surgery, exhibiting satisfactory health.

## Discussion

The timely identification of cornual and interstitial pregnancy is of utmost importance due to its potential to cause life-threatening situations, particularly in countries with limited resources and infrastructure for health care. The clinical manifestation of cornual pregnancy is contingent upon whether the ectopic pregnancy in the cornual region is ruptured or unruptured.

At present, there are no known interventions or therapies that have been shown to be effective in preventing the occurrence of an ectopic pregnancy. Predisposing factors encompass various conditions and circumstances that may contribute to the development of fallopian tube damage [9]. These factors include but are not restricted to inflammatory conditions such as salpingitis and chlamydia, previous tubal ligation, a history of infertility unrelated to tubal disease, ovulation induction procedures, previous ectopic pregnancy, prior tubal surgery, smoking, exposure to diethylstilbestrol, and advanced age [14,15]. Patients who have cornual pregnancies commonly exhibit symptoms at a later stage of gestation, which poses challenges in accurately diagnosing cornual ectopic pregnancies. Haemorrhagic shock is observed in around 25% of patients, hence playing a significant role in the elevated death rate associated with cornual pregnancies [16]. As a result, it has been estimated that around 40% of hysterectomies are performed due to ruptured cornual pregnancies. Additionally, there is a 20% chance of uterine rupture if the pregnancy continues beyond 12 weeks of gestation [16,17]. Historically, the management of interstitial and cornual pregnancy has typically involved laparotomy, cornual resection, or hysterectomy [18].

Nevertheless, in patients who are haemodynamically stable, it may be possible to explore more conservative approaches, such as medical intervention and laparoscopic procedures like laparoscopic cornual resection, laparoscopic cornuostomy, or hysteroscopic removal of interstitial ectopic tissue [18]. Additionally, there have been attempts to address the issue through unilateral uterine artery ligation [19]. According to a prominent institution, it is recommended that methotrexate be employed as the initial course of treatment for women who are haemodynamically stable, experience no pain, have an unruptured ectopic pregnancy, possess a mass less than 35 mm without a visible heartbeat, and exhibit a serum beta-human chorionic gonadotropin (b-hCG) level ranging from 1500 to 5000 mIU/ml [20].

## Conclusions

The diagnosis and treatment of cornual pregnancy pose significant challenges due to the abundant vascular supply in this region and the resemblance of the implanted pregnancy to an eccentrically implanted intrauterine pregnancy. Timely identification of the condition can aid in determining the appropriate management and treatment based on factors such as clinical presentation, haemodynamic stability, serum b-hCG level, and ultrasound results. This case study examines the prompt identification and successful treatment of a ruptured right cornual pregnancy. A prompt decision was made to perform a hysterectomy due to the rupture of the right-sided uterine cornu and the presence of profuse bleeding. No immediate or delayed problems were observed. The primary approach to addressing maternal mortality caused by cornual pregnancy is early detection and intervention. Furthermore, counselling serves a crucial function in mitigating risks associated with subsequent pregnancies.
